# Lack of Fas/FasL Does Not Protect from Latent Herpes Simplex 1 Infection but Decreases Virus-Induced Neurodegeneration

**DOI:** 10.3390/cells14241938

**Published:** 2025-12-05

**Authors:** Magdalena Patrycy, Agnieszka Kauc, Martyna Janicka, Aleksandra Osińska, Andrzej Kowalczyk, Katarzyna Skulska, Małgorzata Antos-Bielska, Marcin Chodkowski, Kristina Eriksson, Małgorzata Krzyżowska

**Affiliations:** 1 Division of Medical and Environmental Microbiology, Military Institute of Hygiene and Epidemiology, 01-163 Warsaw, Poland; magdalena.patrycy@wihe.pl (M.P.); agnieszka.kauc@wihe.pl (A.K.); martyna.janicka@wihe.pl (M.J.); aleksandra.osinska@wihe.pl (A.O.); malgorzata.bielska@wihe.pl (M.A.-B.); marcin.chodkowski@wihe.pl (M.C.); 2 PORT Polish Center for Technology Development, 54-066 Wroclaw, Poland; andrzej.kowalczyk@bioceltix.com (A.K.); kasiamikolajewicz@gmail.com (K.S.); 3 Department of Rheumatology and Inflammation Research, Sahlgrenska Academy, University of Gothenburg, SE-405 30 Gothenburg, Sweden; kristina.eriksson@microbio.gu.se; 4 Department of Pharmaceutical Chemistry and Biomaterials, Faculty of Pharmacy, Warsaw Medical University, 02-097 Warsaw, Poland

**Keywords:** HSV-1, Fas/FasL, neuroinflammation, neurodegeneration, dexamethasone

## Abstract

Many studies have shown an association between herpes simplex virus type 1 (HSV-1) infection and the development of neurodegeneration processes later in life, such as Alzheimer’s disease. The Fas/FasL death pathway plays an important role in the complex regulation of the local inflammatory response and mounting of the specific antiviral response in HSV-1 infection. Here, we applied a mouse model of latent HSV-1 neuroinfection to Fas- and FasL-deficient mice (lpr and gld) to explore whether the lack of functional Fas/FasL pathway protects from inflammation-related neurodegeneration. The latently infected Fas- and FasL-deficient mice (lpr and gld) were not protected from virus replication despite the accumulation of virus-specific cytotoxic T cells. However, the lack of Fas/FasL pathway decreased neuroinflammation- and neurodegeneration-related markers, including cognitive impairment, amyloid-β protein, and tau hyperphosphorylation. The use of a glucocorticoid, dexamethasone, to decrease neuroinflammation in wild-type mice did not protect from cognitive impairment, despite the improved antiviral response. Our data indicate that excessive neuroinflammation via the Fas/FasL pathway during HSV-1 infection is associated with neurodegeneration. Furthermore, the administration of immunomodulatory agents to ameliorate the outcome of HSV-1 latent infection should be restricted to the peak of neuroinflammation.

## 1. Introduction

Herpes simplex virus type 1 (HSV-1) is a DNA virus that establishes a latent infection in sensory ganglia (trigeminal ganglia, TGs) and infected people may expect to have several symptomatic recurrencies within a year, known as cold sores of the orolabial mucosa [1,2]. Since sensory trigeminal neurons are pseudounipolar, HSV-1 can also reach the central nervous system (CNS) via an anterograde transport to the trigeminal nuclei in the brainstem. HSV-1 infection of the CNS due to either primary infection or virus reactivation or re-infection can result in herpes simplex encephalitis (HSE) [1,2,3]. HSE has remarkably poor outcomes despite the availability of good antiviral therapy, and most patients subsequently suffer from neurological sequelae; only 2–5% of patients recover completely [1,2,3]. In the acute phase of HSE, patients demonstrate increased production of proinflammatory interleukin-6 (IL-6) and gamma interferon (IFN-γ), followed by the appearance and long-term persistence of markers of T cell activation and virus-specific antibodies in the cerebrospinal fluid (CSF) [4]. Furthermore, persistent microglial activation lasting over 12 months [5] and white matter lesions resulting from a chronic inflammatory process have also been described, even after a successful antiviral course [6]. Thereafter, the treatment of HSE with acyclovir and adjunctive corticosteroid therapy (dexamethasone or prednisolone) have been described as leading to a significant health improvement [7,8,9].

Many studies have strongly supported the concept of a link between repeated reactivations of HSV-1 and persistent neuroinflammation resulting in neurodegeneration and, eventually, amnestic mild cognitive impairment (aMCI) and Alzheimer’s disease (AD) [10,11,12,13]. Previous studies demonstrated the presence of the HSV-1 genome within the amyloid plaques detected in the brains of AD patients, and, in particular, of those carrying the ε4 allele of apolipoprotein E [14]. Furthermore, the level and avidity index of anti-HSV-1 IgG and IgM (markers of HSV-1 infection and reactivation, respectively) have been correlated with the risk of developing AD [15]. HSV-1 infection in cultured neurons induces the processing of APP as well as the intra- and extra-neuronal accumulation of Aβ and other neurotoxic APP fragments [16]. Mouse brains infected with HSV-1 also show increased deposition of Aβ and hyperphosphylated tau protein during the early phase of infection [17] and cognitive impairment during a latent, recurrent HSV-1 infection [18]. The injection of HSV-1 into the brains of 5xFAD mice overexpressing amyloid induced a rapid increase in Aβ deposition [10]. Therefore, it has been suggested that Aβ accumulation may function as a defense mechanism against HSV-1-related neuroinflammation.

Neuroinflammation is now well recognized as a prominent cause of Alzheimer’s pathology and a potential target for therapy and prevention. Microglia and astroglia are consistently found surrounding amyloid plaques in AD brains [19] but it is believed that microglia can play a dual role in Aβ pathogenesis. While they help to eliminate Aβ aggregates by phagocytosis, at the same time, amyloid deposition causes a microglial-mediated inflammatory response [20]. The Fas receptor (CD95) is known to induce apoptosis when binding to its ligand, FasL (CD178). However, apart from apoptosis induction, Fas fulfils diverse functions in different tissues in vivo [21]. In the CNS, it is involved in axonal outgrowth and adult neurogenesis [22,23], while in the immune system, it mediates survival, proliferation, and inflammation [21]. Fas signaling pathways induce both classical apoptotic pathways, involving the activation of caspases, and non-apoptotic pathways, such as NF-κB, MAPK, and PI3K/AKT pathways, albeit through different mechanisms [21,24]. Non-apoptotic pathways can regulate immune responses and promote cell proliferation, migration, and invasion by inducing the production of inflammatory factors and chemokines [21,24]. Furthermore, even apoptotic death during infection is not totally silent in terms of produced inflammatory cytokines and chemokines, and it often becomes a target of pathogen-encoded subversion [21,24].

In the brains of AD patients, both Fas and FasL proteins were identified in amyloid plaques and neurofilament-positive dystrophic neurites and were associated with caspase activation and apoptosis [25]. Furthermore, certain Fas gene polymorphisms were identified as increasing the risks of developing AD and other dementia types [26]. However, little is known about the role of Fas/FasL in the neuroinflammation induced by HSV-1 infection or in the context of infection-induced neurodegeneration.

We have previously demonstrated that HSV-1 infection induces the infiltration of Fas- and FasL-bearing monocytes and T cells in the brain, but it also upregulates Fas and FasL expression in resident astrocytes and microglia within infected sites [27]. The lack of Fas or FasL partially protects from encephalitis with decreased morbidity and mortality compared to WT mice, indicating that Fas/FasL deficiency promotes cell-mediated immunity within the CNS [27,28]. In the brains of HSV-1-infected mice, infected sites were surrounded by FasL expressing microglia [27,28]. Furthermore, we identified activated FasL-bearing microglia surrounding HSV-1-infected sites with deposition of Aβ [28].

The main aim of this study is to elucidate the role of Fas/FasL in HSV-1-induced neuroinflammation and AD-related brain pathologies such as the deposition of Aβ and phosphorylated tau proteins. Furthermore, we also address the possible positive effects of corticosteroid therapy (dexamethasone) in reducing HSV-1-related neuroinflammation and neurodegeneration.

## 2. Materials and Methods

### 2.1. Virus and Cells

HSV-1 (strain ID 2762) was obtained from a clinical case of severe herpesvirus encephalitis (kindly provided by Professor Thomas Bergström, Department of Virology, University of Gothenburg). It was grown and titrated in Vero 76 cells (ATCC^®^ CRL-1587, ATCC, Washington, DC, USA, as described previously [27]). Mixed glial and microglial cultures were obtained and grown as described by Krzyzowska et al. [27]. Microglial cell cultures were used within 48 h of harvesting.

### 2.2. Mice and Infection

Female and male (50:50) 6- to 8-week-old C57BL/6 B6. MRL-Fas lpr/J (Fas−) and B6Smn.C3-Fasl gld/J (FasL−) mice were purchased from Charles River (Dortmund, Germany) and bred at the animal facility of the Mossakowski Medical Research Institute Polish Academy of Sciences (MMRI PAS). The experiments obtained approval from the 2nd Animal Research Ethical Committee in Warsaw (permission nos. WAW2/068/2021, WAW2/008/2024), and animal experimentation guidelines were strictly followed. Details of the animal studies have been described previously [27,29]. Dexasone (ScanVet, Gniezno, Poland) at 2 mg/mL was administered at 0.2 mg/mouse daily via an intraperitoneal treatment. The tissues for further tests were collected at 7 and 120 days post-infection (p.i.).

### 2.3. Flow Cytometry Analysis

The brains and trigeminal ganglia of HSV-1-infected and uninfected control mice were used to prepare single-cell suspensions, as described previously [27,29,30]. Details of the staining procedure, gating strategy, and antibody clones were described previously [27,29,30]. HSV-1-specific T cells were detected using the SSIEFARL-PE tetramer (Creative Biolabs, Shirley, NY, USA). Stained cell suspensions were acquired using CytoFLEX LX (Beckman Coulter, Warsaw, Poland) and analyzed using FlowJo software V10.10 (Tree Star, Ashland, OR, USA).

### 2.4. Quantitative PCR

Isolation of the total DNA and RNA from the brains and trigeminal ganglia and titration of HSV-1 were performed as described previously [27,29] using the QuantStudio™ 5 Real-Time PCR System (Thermo Fisher Scientific, Waltham, MA, USA) with GoTaq^®^ Probe qPCR Master Mix (Promega, Madison, WI, USA). Data were calculated as the HSV-1 copy number per ng of the total DNA in the tissue. The titration of immediate early gene ICP0, a leaky-late gene (gB) and latency-associated transcript (LAT), as well as the expression of cytokines and chemokines were described before [27,29]. The results were analyzed and presented with the 2^−ΔΔCt^ method (2^−ΔΔCt^).

### 2.5. Confocal Microscopy

Brains fixed in 4% paraformaldehyde/PBS were saturated with 30% sucrose/PBS, and then frozen and cut into cryostat sections [27,29]. The sections were stained overnight at 4 °C as described previously using the following primary antibodies: rabbit polyclonal anti-HSV-1/2 (Dako, Agilent, Santa Clara, CA, USA), goat polyclonal anti-NeuN (Thermo Fisher Scientific), and mouse monoclonal anti-β-Amyloid-biotin (clone 12F4, BioLegend, San Diego, CA, USA) [24,26]. Images were acquired using a Zeiss Laser Scanning Inverted Microscope LSM-700 (Carl Zeiss, Jena, Germany) equipped with 40×/1.3 Oil NA objective and Zeiss Zen 3.13. software (Carl Zeiss).

### 2.6. Behavioral Tests

The Novel Object Recognition (NOR) test was a three-day procedure consisting of (i) a familiarization phase, (ii) a training phase, and (iii) a test phase, each lasting 10 min. During the familiarization phase (day 1), the mice were individually submitted into the empty arena (45 × 45 cm). On the 2nd day (training phase), the animals were allowed to explore two identical objects placed in a symmetric position in the arena center (two plastic blocks). Explorative behavior was scored when the head of the animal was close to the object or any part of the body except the tail was touching the object. We recorded the time spent exploring each object. On the 3rd day, during the test phase, one of the objects used during the training was replaced by a novel object (a different plastic block) and the animals were allowed to explore freely for 10 min. The preference index, i.e., the ratio of the amount of time spent exploring any one of the two items or the novel object over the total time spent exploring both objects, was used to measure recognition memory.

### 2.7. ELISA

Brain homogenates were prepared and quantified according to the ELISA kit manufacturer’s instructions: Mouse Amyloid Beta 42 (Ms Abeta 42) (Thermo Fisher Scientific), and Mouse Tau (Phospho) [pS199] (Thermo Fisher Scientific). The values of amyloid beta and mouse tau proteins were normalized to total mass organ and total protein concentration.

### 2.8. Mouse Tight Junction PCR Array

Total RNA was isolated as described above and used for cDNA synthesis with the RT2 PCR Array First Strand kit (Qiagen, Hilden, Germany). The expression of 84 genes involved in tight junctions and four housekeeping genes (Beta actin; Gadph, glyceraldehyde-3-phosphate dehydrogenase; Hsp90ab1, heat shock protein 90 alpha; and Gusb, glucuronidase beta) was measured with RT^2^ Profiler™ PCR Array Mouse Tight Junctions arrays (PAMM-143Z; Qiagen) containing pre-developed reactions, according to the manufacturer’s instructions, the in Quant Studio™ 5 Real-Time PCR System (Thermo Fisher Scientific). Data analysis was performed using GeneGlobe (Qiagen) PCR array data analysis software available online. To determine fold change in gene expression, the normalized expression of the gene of interest (GOI) at 7 days of HSV-1 infection was divided by the normalized expression of the same GOI in the uninfected control sample.

### 2.9. Statistics

All statistics was performed using GraphPad Prism version 7 (GraphPad software). Before further analysis, the data were first subjected to the Shapiro–Wilk test for normality and Levene’s test for equality of variances. The nonparametric Wilcoxon test for dependent samples was applied to the data with non-Gaussian distributions, while the Kruskal–Wallis test with post hoc multiple comparisons was used for the comparison of all pairs (infected vs. uninfected; primary infection vs. latent infection). Box sexes were included in the analysis. The results are reported as the mean ± standard error of the mean (SEM). A value of *p* < 0.05 was considered statistically significant.

## 3. Results

### 3.1. Lack of Fas/FasL Does Not Protect from Latent HSV-1 Infection

**Figure 1:**
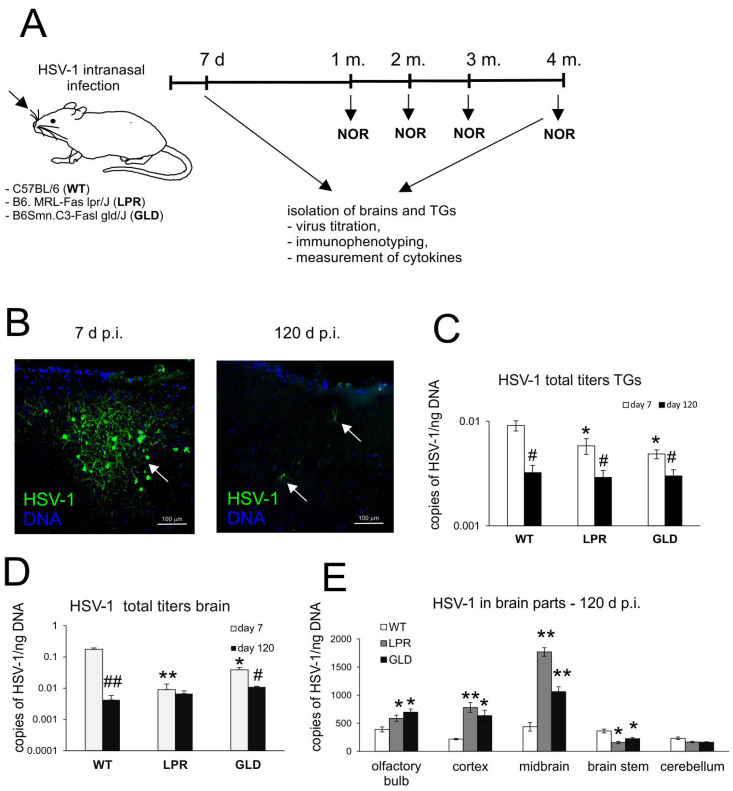
Lack of Fas or FasL does not protect from latent HSV-1 infection. (**A**) Schematic representation of experimental design. (**B**) Immunofluorescent staining for HSV-1 antigens (green) in the brains of mice infected for 7 and 120 days. White arrows indicate HSV-1-positive cells. Nuclei were counterstained for DNA with DAPI (blue). Magnification ×200. (**C**–**E**) HSV-1 titers were defined as gB gene copies/ng DNA in TGs (**C**) for whole brains (**D**) and brain parts—olfactory bulb, cortex, brainstem, midbrain, and cerebellum—(**E**) at 7 and 120 d p.i. by qPCR. C57BL/6 (WT), B6. MRL-Fas lpr/J (LPR) and B6Smn.C3-Fasl gld/J (GLD) mice were infected intranasally with HSV-1, as described in the Materials and Methods section. Results presented as the mean ± SEM. N = 7. # Represents significant differences with *p* ≤ 0.05, and ## means *p* ≤ 0.01 in comparison to 7 days p.i. (latent vs. primary infection), while * represents significant differences with *p* ≤ 0.05, and ** *p* ≤ 0.01 in comparison to the wild-type (WT) infected tissues (GLD or LPR vs. WT).

We demonstrated previously that mice lacking Fas or FasL expression are protected from early HSV-1 infection of the brain [27]. To further check whether this protection can last during latent infection, we compared viral titers in C57BL/6 (WT), B6. MRL-Fas lpr/J (Fas−) (LPR) and B6Smn.C3-Fasl gld/J (FasL−) (GLD) mice in primary infection (7 d p.i.) and in latent infection (120 d p.i.) (Figure 1A). We showed that while the WT C57BL/6 mice were more susceptible to early HSV-1 infection within first 30 days and their mortality rate reached plateau at month 2, LPR and GLD mice died later, reaching similar numbers of deceased animals at the end of the study (Supplementary Figure S1). The determination of viral titers in the brains and trigeminal ganglia (TGs) at both 7 and 120 d p.i. (Figure 1) showed that viral titers in TGs and brains significantly decreased at 120 d p.i. independently of the infected mouse strain (*p* ≤ 0.05) (Figure 1C). However, the brains of mice with all tested strains showed similar HSV-1 titers (Figure 1D) at 120 d p.i. These HSV-1 titers contrasted with early infection, when LPR and GLD mice showed significantly lower viral titers in all tested organs (Figure 1). We further tested HSV-1 titers in different brain parts at 120 d p.i. (Figure 1E). The olfactory bulbs, cortex, and midbrain of LPR and GLD mice demonstrated significantly higher HSV-1 titers compared to wild-type mice (*p* ≤ 0.05) (Figure 1D), while the brain stems contained significantly less HSV-1 (*p* ≤ 0.05) (Figure 1D).

**Figure 2:**
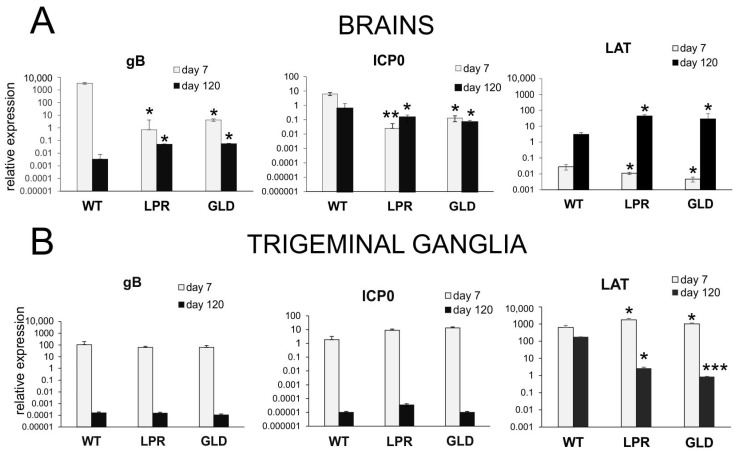
Lack of Fas or FasL influences latency-related transcripts. gB, ICP0 and LAT mRNA viral transcripts were measured by qPCR in brains (**A**) and TGs (**B**), collected at 7 and 120 days p.i. (N = 7). C57BL/6 (WT), B6. MRL-Fas lpr/J (LPR), and B6Smn.C3-Fasl gld/J (GLD) mice were infected intranasally with HSV-1, as described in Materials and Methods Section 2. The bars represent means ± SEMs. * Represents significant differences with *p* ≤ 0.05 and ** means *p* ≤ 0.01 while *** means *p* ≤ 0.001 in comparison to the wild-type (WT) infected tissues at 7 or 120 d p.i. (GLD or LPR vs. WT).

The expressions of lytic/latency-related genes in the brains of LPR and GLD mice were significantly decreased for all tested viral genes at 7 d p.i., while during latency, the expression of mRNA for the lytic gene gB and latency-associated transcript (LAT) increased significantly compared to wild-type mice (*p* ≤ 0.05) (Figure 2A). The expression of reactivation-related ICP0 mRNAs significantly decreased during latency in LPR and GLD mice (*p* ≤ 0.05) (Figure 2A). For TGs, LPR and GLD mice showed a significant increase in LAT mRNAs during early stage of infection and subsequently decreased in latency compared to wild-type mice (*p* ≤ 0.05) (Figure 2B).

**Figure 3:**
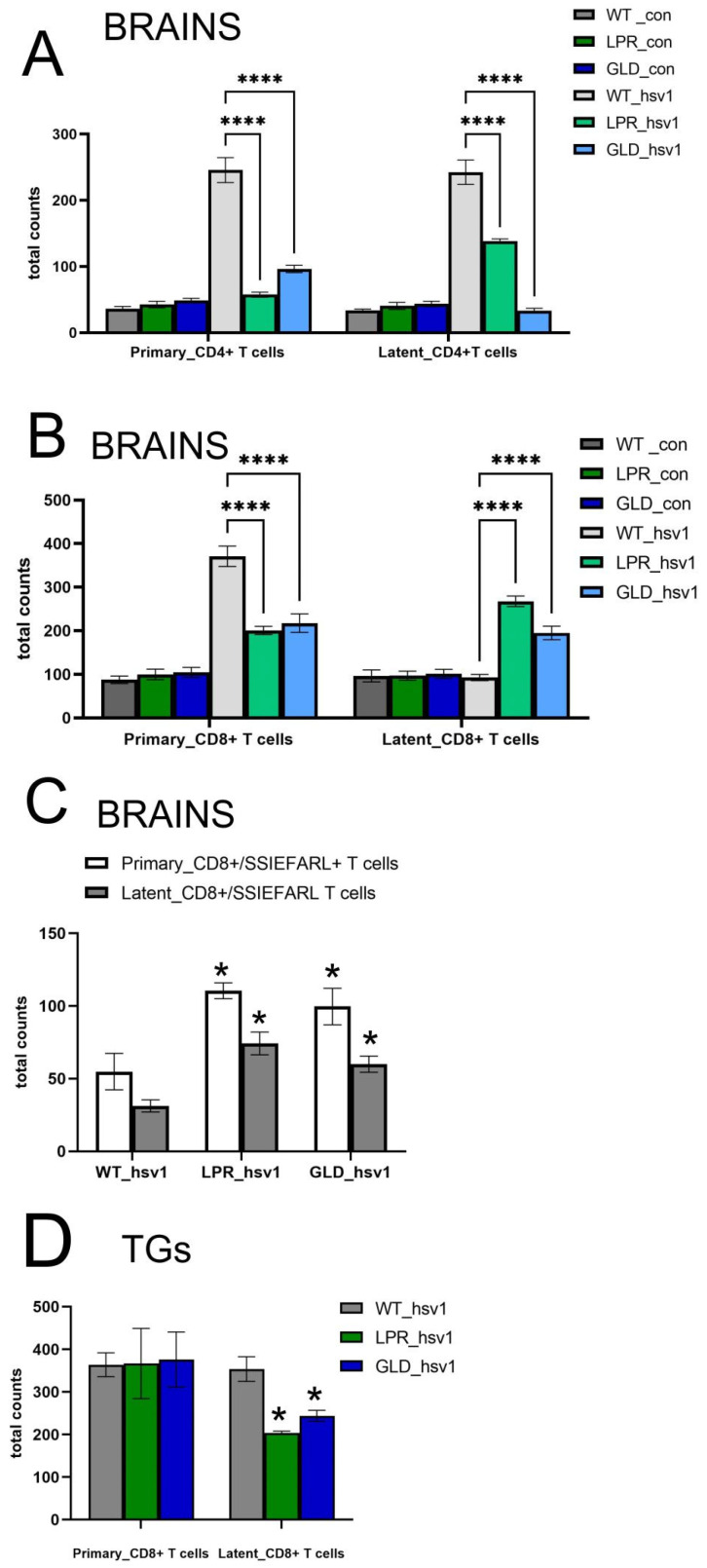
Fas/FasL influences the infiltration of T cells during latent HSV-1 infection. Cell counts for CD4+ T cells (**A**), CD8+ T cells (**B**), and CD8+/SSIEFARL+ T cells (**C**) in brains and CD8+ T cells (**D**) in TGs measured by flow cytometry in uninfected (con) and infected (hsv-1) mice at 7 (primary) and 120 (latent) days p.i. C57BL/6 (WT), B6. MRL-Fas lpr/J (LPR), and B6Smn.C3-Fasl gld/J (GLD) mice were infected intranasally with HSV-1, as described in the Materials and Methods section. Data presented as mean ± SEM, N = 7. **** Represents significant differences with *p* ≤ 0.001 and * *p* ≤ 0.05. Data analysis was performed by comparing LPR and GLD mice with WT mice.

To compare the antiviral response in LPR and GLD mice during primary infection and latency, we counted the numbers of CD4+ T cells and CD8+ T cells in the brains and TGs at 7 and 120 days of infection using flow cytometry (Figure 3). We observed that LPR and GLD mice showed significantly lower numbers of CD4+ T cells during both early and latent infection compared to wild-type mice (*p* ≤ 0.001) (Figure 3A). We found that while the numbers of CD8+ T cells were significantly lower in LPR and GLD mice during early infection (*p* ≤ 0.001) (Figure 3B), these mice showed significantly higher numbers of CD8+ T cells during latent infection (*p* ≤ 0.001) (Figure 3B). Interestingly, during both early and latent infection, LPR and GLD mice demonstrated significantly higher counts of HSV-1-specific cytotoxic CD8+ T cells/SSIEFARL+ compared to the wild-type mice (*p* ≤ 0.05) (Figure 3C). For TGs, we observed significantly lower numbers of CD8+ T cells during latent infection in LPR and GLD mice (*p* ≤ 0.05) (Figure 3D), but no differences for CD8+ T cells/SSIEFARL+.

Microglia and infiltrating monocytes play an important role in the neuroinflammation observed during HSV-1 infection. Here, the numbers of infiltrating monocytes in both brains (Supplementary Figure S2A) and TGs (Supplementary Figure S2A) were significantly lower in LPR and GLD mice during early infection and only in latent TGs (*p* ≤ 0.001) (Supplementary Figure S2A,B). The latent brains of LPR and GLD mice did not differ in numbers of microglia or infiltrating monocytes from uninfected controls or infected wild-type mice (*p* ≤ 0.001) (Supplementary Figure S2A,C).

Antiviral and/or inflammatory cytokines/chemokines produced by microglia, astrocytes, and other infiltrating immune cells contribute to the neuroinflammation observed during HSV-1 infection of the CNS [24]. Here, we observed that latently infected LPR and GLD mice showed significantly lower expression of mRNA for antiviral CXCL9, CXCL10, and IFN-γ compared to WT mice (*p* ≤ 0.05) (Supplementary Figure S3). No significant differences between latently infected strains were observed for the inflammatory cytokines IL-1β and TNF-α (*p* ≥ 0.05) (Supplementary Figure S3).

### 3.2. Lack of Fas/FasL Protects from Neurodegeneration in Latent HSV-1 Infection

**Figure 4:**
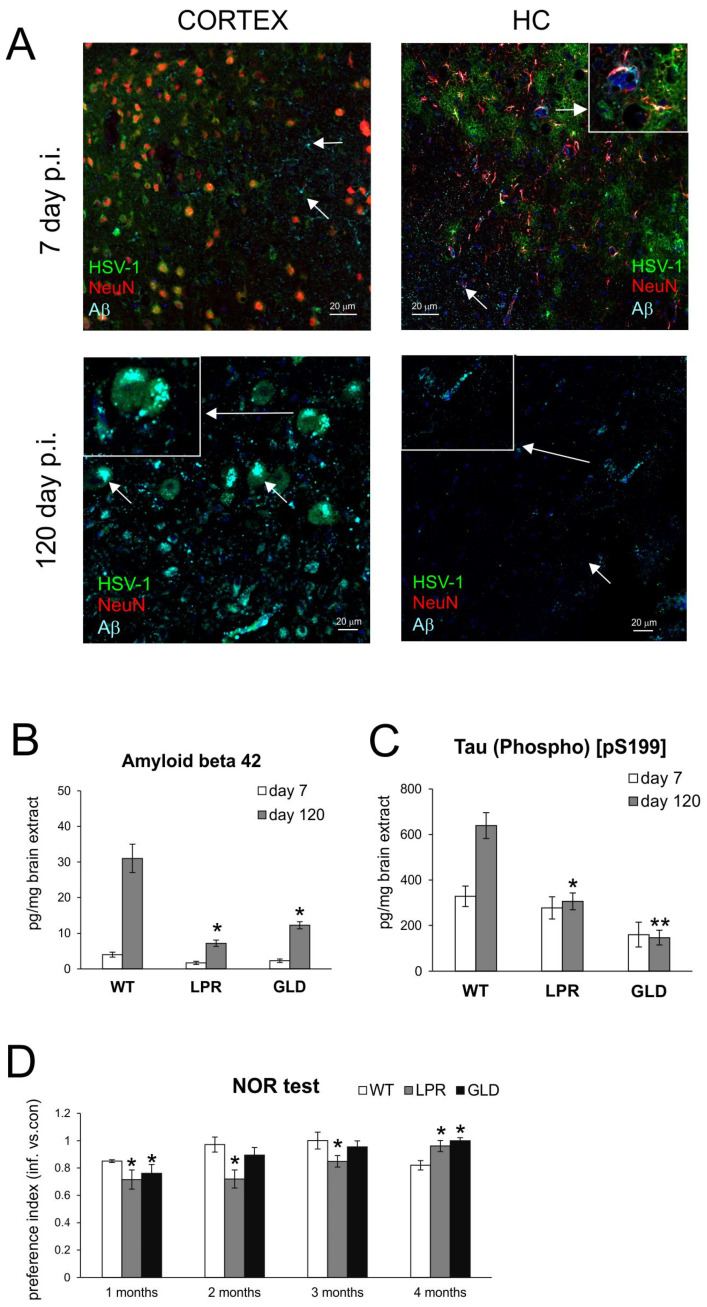
Lack of Fas/FasL protects from neurodegeneration in latent HSV-1 infection. (**A**) Confocal immunofluorescence images of β-amyloid (Aβ) (turquoise) and NeuN (red) expression in brains of HSV-1-infected mice at 7 and 120 d p.i. Representative images of different areas within somatosensory neocortex and hippocampus (HC) are shown. White arrows indicate sites of β-amyloid accumulation. Nuclear bodies were stained by DAPI (blue), magnification ×200. (**B**,**C**) Beta amyloid 1–42 (**B**) and tau (Phospho) [pS199] (**C**) levels in brain homogenates by ELISA. (**D**) Results of NOR tests expressed as mean values of preference index (PI) for novel objects at 1, 2, 3, and 4 months from infection. C57BL/6 (WT), B6. MRL-Fas lpr/J (LPR), and B6Smn.C3-Fasl gld/J (GLD) mice were infected intranasally with HSV-1, as described in the Materials and Methods section. Data analysis was performed by comparing Fas-deficient (lpr) and FasL-deficient (gld) groups with wild-type (C57BL/6) mice. The bars represent means ± SEMs. ** *p* ≤ 0.01, and * *p* ≤ 0.05.

Next, to check whether the lack of Fas or FasL can also protect animals from neuropathological changes induced by latent HSV-1 infection, we checked the appearance of Aβ (beta-amyloid), phosphorylated tau, and behavioral changes in mice during primary and latent infection (Figure 4). An immunofluorescence analysis of confocal microscope slides of brain slices showed the presence of Aβ during early infection in the somatosensory cortex and hippocampus within the area surrounding HSV-1 infectious foci (Figure 4A). During latent infection, greater Aβ accumulation was found in the somatosensory cortex compared to the hippocampus, and Aβ accumulation was detected as intra- and extracellular deposits around latently infected neurons (Figure 4A). Next, we compared levels of Aβ and phosphorylated tau (pS199) in brain extracts from mice in primary and latent infection (Figure 4B,C). While the mice from all tested strains did not differ in levels of Aβ or tau during primary infection, mice lacking Fas or FasL demonstrated significantly lower levels of these proteins in the latent HSV-1 infection (*p* ≤ 0.05) (Figure 4B,C).

Next, we checked whether the lack of Fas or FasL in mice influences the impairment of cognitive functions in mice latently infected with HSV-1. To address this issue, mice infected with HSV-1 and the age-matched controls were tested using the Novel Object Recognition test (NOR), which is commonly used to assess hippocampal-dependent learning and memory. Following the first 4 weeks after the primary infection, the mice were tested once a month (Figure 1 and Figure 4D). At the first month of infection, HSV-1-infected mice of all strains showed a significant decrease in the preference index (PI) compared to controls (*p* ≤ 0.05) (Figure 4D). During next two months, only Fas-lacking mice (LPR) showed significantly decreased PI (*p* ≤ 0.05) (Figure 4D). At 120 d p.i., LPR and GLD mice showed no cognitive impairment induced by HSV-1 primary infection, indicating recovery (Figure 4D). On the other hand, wild-type mice demonstrated a significant decrease, at the level observed after 30 days of infection (Figure 4D), thus suggesting that the cognitive impairment induced by HSV-1 latent infection of WT mice increases over time.

We previously demonstrated that the Fas/FasL pathway leads to excessive neuroinflammation during HSV-1 infection, accompanied by a diminished antiviral response. Although HSV-1 enters the brain via a neuronal route, the damage of the BBB in HSE results in aggravated leukocyte infiltration. To understand whether Fas/FasL expression can contribute to the tightness of the BBB, we used a PCR array to study the expression of tight junction proteins involved in the BBB, such as claudins, occludin, zona occludens (ZO) proteins, junctional adhesion molecules (JAMs), ESAM, endothelial-cell-selective adhesion molecules, and others. The analysis showed that HSV-1 infection of the wild-type brains induced a significant increase in RNA expression for claudins (1, 4, 5, 6, 15, and 16), ESAM, JAM-1, and hematopoietic-cell-specific Lyn substrate 1 (Hcls1) (*p* ≤ 0.05) (Supplementary Table S1), but it had no influence upon the expression of other tight-junction-related proteins. The expressions of tight-junction-related genes were also upregulated during the HSV-1 infection of LPR and GLD mice, albeit insignificantly. Furthermore, expressions of claudin 1, 4, 6, 8, 15, 16, and 18 in HSV-1-infected LPR and GLD mice were significantly decreased compared to infected wild-type mice (*p* ≤ 0.05) (Supplementary Table S1).

### 3.3. Dexamethasone Treatment During Primary Infection Does Not Protect from Neurodegeneration in Latent Infection

**Figure 5:**
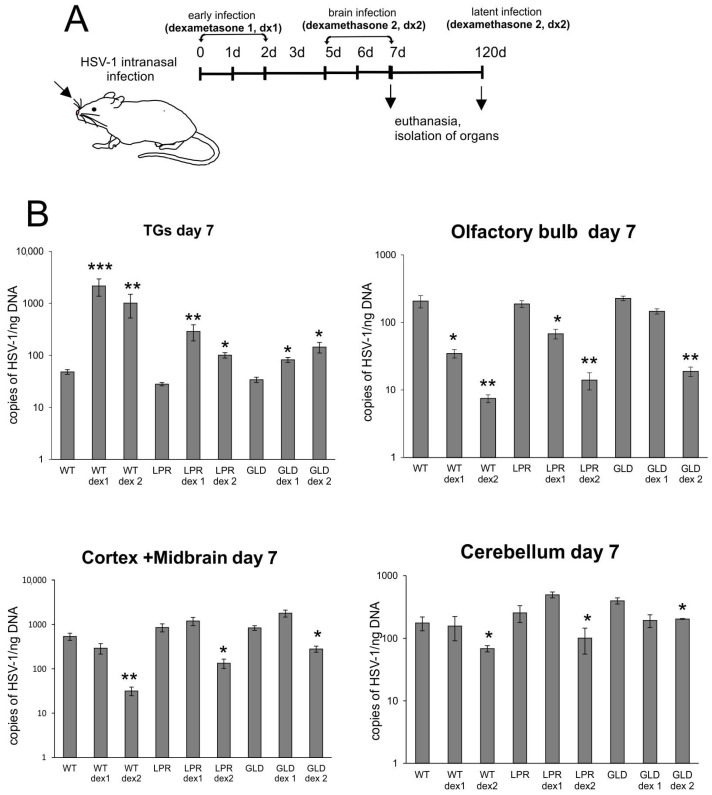
Dexamethasone treatment during primary infection decreases HSV-1 titers in brains but not in TGs. (**A**) Schematic representation of experimental design. (**B**) HSV-1 titers measured as gB gene copies/ng DNA measured in TGs, and brain parts—olfactory bulb, cortex/midbrain, and cerebellum—at 7 d p.i. by qPCR. C57BL/6 (WT), B6. MRL-Fas lpr/J (LPR), and B6Smn.C3-Fasl gld/J (GLD) mice were infected intranasally with HSV-1, and treated with dexamethasone on day 1 and 2 (DEX 1) or day 5 and 6 (DEX2) post infection. Results expressed as the mean ± SEM for N = 7. * Represents significant differences with *p* ≤ 0.05, ** *p* ≤ 0.01, *** *p* ≤ 0.001 in comparison to untreated infected mice.

Dexamethasone treatment is frequently used in HSE in conjunction with acyclovir to protect patients from the development of autoaggressive antibodies and excessive neuroinflammation. Here, to confirm whether dexamethasone treatment can indeed protect from neuroinflammation and HSE-related neurological sequelae, we treated HSV-1 early during infection (DEX1), or later, during the peak of brain infection, when neurological symptoms are already present (DEX2) (Figure 5A). Mice treated early with dexamethasone (DEX1) showed significantly increased mortality compared to untreated infected mice—the survival rate at 7 d p.i. for wild-type mice was 55 ± 2% vs. 78 ± 3%, while for Fas-deficient (LPR) and FasL-deficient (GLD) mice it was 75 ± 4% vs. 95 ± 3%, and 80 ± 2% vs. 93 ± 2%, respectively. When dexamethasone was given at the peak of infection, we observed the opposite results for wild-type mice (91 ± 2% vs. 78 ± 3% survived mice), while for LPR and GLD mice, the differences between treated and untreated mice were insignificant. Furthermore, we measured the viral titers in the TGs and brain parts—olfactory bulbs, midbrain with cortex, and cerebellum—at 7 d p.i. and found that dexamethasone treatment significantly increased HSV-1 titers in TGs isolated from all tested strains, irrespective of the treatment regime (DEX1 or DEX2) (*p* ≤ 0.05) (Figure 5B). For the olfactory bulbs, both dexamethasone treatment regimens significantly decreased HSV-1 titers (*p* ≤ 0.05) (Figure 5B), while for cortex, midbrain, and cerebellum, only DEX2 treatment at the peak of infection significantly decreased HSV-1 titers (*p* ≤ 0.05) (Figure 5B).

**Figure 6:**
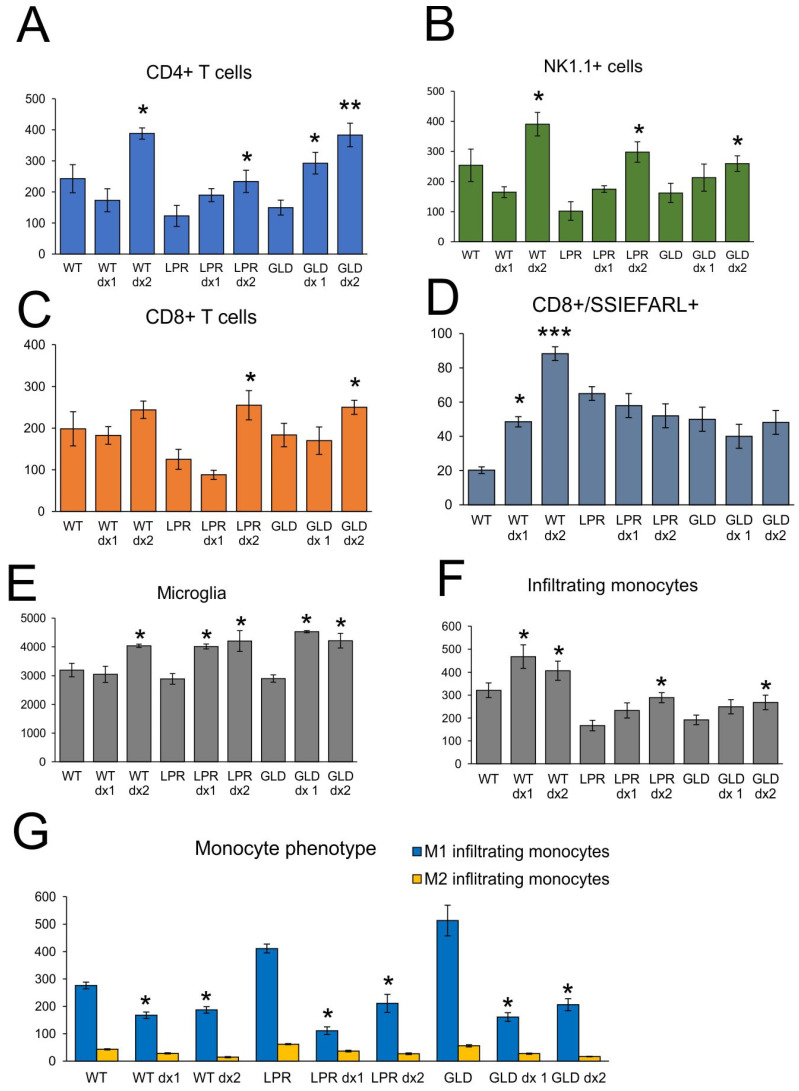
Dexamethasone influences the infiltration of immune-competent cells during primary HSV-1 infection. CD4+ T cells (**A**), NK-1 cells (**B**), CD8+ T cells (**C**), CD8+/SSIEFARL+ T cells (**D**), microglia (**E**), inflammatory monocytes (**F**), and (**G**) M1/M2 monocytes in brains measured by flow cytometry in mice infected at 7 days p.i. C57BL/6 (WT), B6. MRL-Fas lpr/J (Fas−), and B6Smn.C3-Fasl gld/J (FasL−) mice were infected intranasally with HSV-1 and treated with dexamethasone on day 1 and 2 (DEX 1) or day 5 and 6 (DEX2) post infection. Results expressed as the mean ± SEM for N = 7. *** *p* ≤ 0.001, ** *p* ≤ 0.01, and * *p* ≤ 0.05 compared to untreated infected mice.

To further understand how dexamethasone influences an early antiviral response in HSV-1 infection, we measured the counts of NK cells, CD4+ T cells, CD8+ T cells, and HSV-1-specific CD8+/SSIEFARL+ T cells in the brains at 7 days of infection by flow cytometry (Figure 6). We observed that DEX2 treatment significantly increased CD4+ T cells and NK cells in all tested mouse strains (*p* ≤ 0.05) (Figure 6A,B) and CD8+ T cells only in LPR and GLD mice (*p* ≤ 0.05) (Figure 6C). Interestingly, wild-type mice treated with DEX1/2 regime showed significantly increased numbers of HSV-1 specific cytotoxic T cells compared to untreated mice (*p* ≤ 0.05) (Figure 6D). Furthermore, upon DEX2 treatment, the numbers of microglia and monocytes increased in a significant manner (*p* ≤ 0.05) (Figure 6E,F). DEX1 treatment also increased the number of microglia in LPR and GLD mice and inflammatory monocytes in wild-type mice (*p* ≤ 0.05) (Figure 6E,F). Further measurements of monocytes with active M1 or inti-inflammatory phenotype M2 showed that DEX treatment decreased numbers of pro-inflammatory M1 monocytes in all tested strains (*p* ≤ 0.05) (Figure 6G).

**Figure 7:**
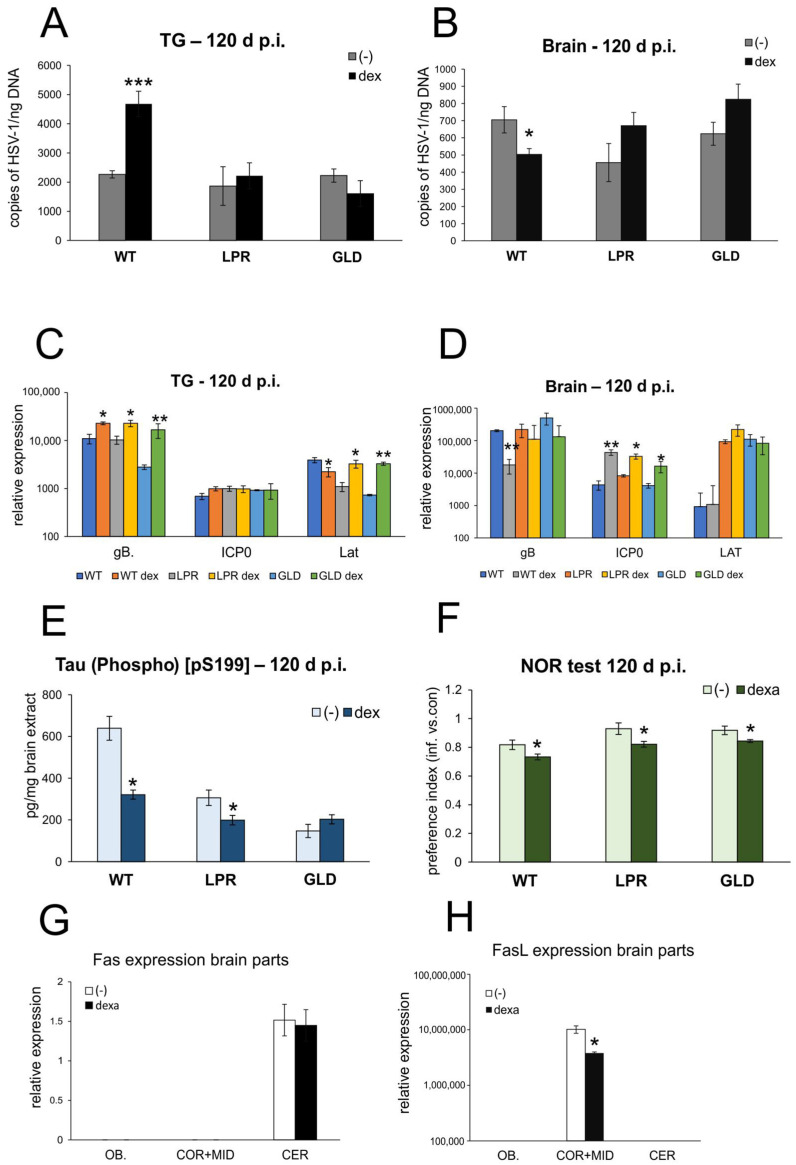
Dexamethasone treatment during primary infection does not protect from neurodegeneration in latent infection. HSV-1 gB titers (copies/ng DNA) measured in trigeminal ganglia (TGs) (**A**) and brains (**B**) at 120 d p.i. by qPCR. Expression of gB, ICP0, and LAT viral transcripts in TGs (**C**) and brains (**D**) collected at 120 days p.i., measured by qPCR. (**E**) Tau (Phospho) [pS199] concentration in brain homogenates by ELISA. (**F**) Mean values of preference index (PI) for novel object. Fas (**G**) and (**H**) FasL expression in brain parts measured by qPCR. C57BL/6 (WT), B6. MRL-Fas lpr/J (Fas−), and B6Smn.C3-Fasl gld/J (FasL−) mice were infected intranasally with HSV-1 and treated with dexamethasone on day 5 and 6 (DEX2) post infection. At day 120, brains and TGs were collected for further studies. Before sacrifice, mice were subjected to Novel Object Recognition (NOR) test as described in the Materials and Methods section. Results expressed as the mean ± SEM for N = 7. *** *p* ≤ 0.001, ** *p* ≤ 0.01, and * *p* ≤ 0.05 compared to untreated infected mice.

Considering previous results showing that neurodegenerative changes accumulate in wild-type HSV-1-infected mice with time, we tested whether dexamethasone treatment during the peak of brain infection (DEX2) can influence this process in latency. The detection of HSV-1 copies in latently infected mice at 120 d p.i. demonstrated that DEX2 treatment significantly increased HSV-1 titers in TGs (*p* = 0.001) (Figure 7A), while it decreased viral replication in brains isolated from wild-type mice (*p* = 0.04) (Figure 7B). DEX2 treatment increased HSV-1 titers in the brains isolated from LPR and GLD mice, albeit insignificantly (Figure 7B). Furthermore, DEX2 treatment increased gB mRNA expression in TGs in all mice strains (*p* ≤ 0.05) (Figure 7C), but had no influence upon reactivation-related ICP0 (Figure 7C). For latency-related transcripts (LAT), DEX2 treatment decreased the expression of LAT in the TGs of DEX2-treated wild-type mice, and increased it in LPR and GLD mice (*p* ≤ 0.05) (Figure 7C). In the latently infected brains, DEX2 treatment had no influence upon LAT (Figure 7D), but it significantly increased ICP0 expression (*p* ≤ 0.05) (Figure 7D). DEX2 treatment also had no influence upon previously observed Aβ levels, but decreased tau levels in latently infected wild-type and Fas(−) mice (*p* ≤ 0.05) (Figure 7E). Interestingly, dexamethasone treatment decreased the PI compared to untreated mice at 120 d p.i., indicating cognitive impairment in DEX-treated mice, irrespective of the tested strain (*p* ≤ 0.05) (Figure 7F). We also measured the expression of Fas and FasL in different brain parts in latency (Figure 7G,H), showing that Fas expression was detected only in the cerebellum, while FasL expression was found in the cortex, with DEX2 causing a significant decrease (*p* = 0.048) (Figure 7H).

**Figure 8:**
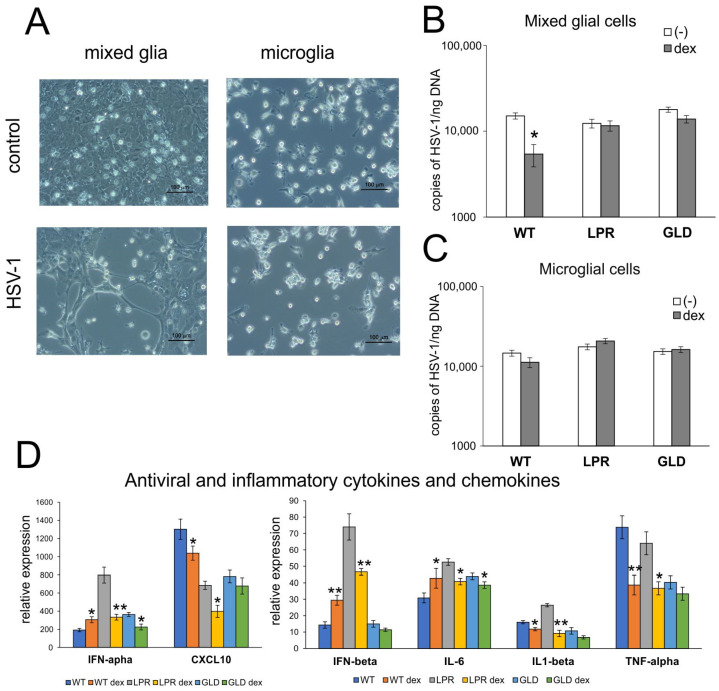
Dexamethasone influences HSV-1 replication only in wild-type glial cells. (**A**) Representative microphotographs of uninfected (upper) and HSV-1-infected (bottom) mixed glial (left) and microglial (right) cultures at 24 h p.i. Titers of HSV-1 gB copies/ng DNA measured in mixed glial (**B**) and microglial (**C**) cells treated (dex) and untreated (-) with dexamethasone (10 μM) at 24 h p.i. (**D**) Expression of IFN-α, IFN-β, CXCL10, IL-6, TNF-α, and IL-1β at 24 h p.i. in mixed glial cells treated and untreated with dexamethasone (10 μM) by qPCR. Each bar represents the mean from 3 experiments (N = 3) ± SEMs; * represents significant differences with *p* ≤ 0.05 while ** signifies *p* ≤ 0.01 in comparison to infected untreated cells.

To understand how dexamethasone can influence the HSV-1 infection of glial cells and early antiviral response, we used a primary culture of mixed glial cells and microglial cells (Figure 8). We found that dexamethasone decreased HSV-1 replication in mixed glial cells prepared from WT mice (*p* = 0.049) (Figure 8B), but had no effect upon HSV-1 replication in microglial cells (Figure 8C). Dexamethasone treatment also decreased the expression of mRNA for IFN-α, IFN-β, CXCL10, IL-6, IL-1β, and TNF-α in HSV-1-infected mixed glial cultures prepared from Fas- or FasL-deficient mice (*p* ≤ 0.05) (Figure 8D). However, dexamethasone increased mRNA for IFN-α, IFN-β, and IL-6, but decreased mRNA for CXCL10, IL-1β, and TNF-α in wild-type mixed glial cells (Figure 8D).

## 4. Discussion

Our present study discusses the role of Fas/FasL in HSV-1 latent infection as a pathway involved in neuroinflammation and antiviral response. Here, we also provide novel evidence in mice that treatment with a corticoid drug—dexamethasone—does not protect mice from latent infection and latency-related neurodegeneration.

The role of the Fas/FasL pathway in maintaining the immune homeostasis and elimination of infected cells via apoptosis induction has been widely studied and discussed [21,27]. We previously demonstrated that, in the HSV-1 brain infection, the upregulation of Fas and FasL expression correlated with HSV-1 infection in a space- and time-dependent manner, and the main source of Fas-FasL interactions within the HSV-1-infected brain came from infiltrating monocytes and T cells [27]. When employing the HSV-1 infection model to Fas- and FasL-deficient (lpr and gld) mice, we detected reduced morbidity and mortality compared to wild-type mice resulting from significantly lower virus replication in the brains and trigeminal ganglia in primary infection [27]. Furthermore, a lack of Fas or FasL expression actually led to better infiltration of HSV-1-specific cytotoxic T cells [27]. Here, we employed the same model to determine whether a lack of Fas or FasL can protect against latent infection with HSV-1. Compared to early primary infection (day 7), mice lacking Fas or FasL in latent infection (120 d p.i.) did not differ in mortality or virus titers from HSV-1-infected wild-type mice. Surprisingly, viral titers in mice lacking Fas or FasL were only slightly decreased in latency compared to the primary infection in both brains and TGs. Relatively high viral titers in latently infected brains were also followed by the increased expression of gB and LAT viral transcripts compared to wild-type mice, altogether indicating insufficient control of HSV-1 reactivation/latency in Fas- and FasL-deficient mice in a long-term infection. During latency in mice, CD8+ T-cells specific for HSV are described to be closely localized around the infected neurons and form immunological synapses [31]. Here, Fas- or FasL-deficient mice showed significantly higher numbers of CD8+/SSIEFARL+ T-cells specific for HSV antigens in latency compared to wild-type mice. Yajima et al. [32] found that during a prolonged tumor immune response, Ag-specific activated CD8+ T cells died via Fas-FasL apoptosis. This may suggest that the accumulation of CD8+/SSIEFARL+ T-cells in primary and latent HSV-1 infection of the brain and TGs in mice lacking Fas or FasL results from a lack of apoptosis of virus-specific CD8+ T cells. However, these cells may not be efficient in controlling HSV-1 brain infection; HSV-1-specific CD8 T-cells have been shown to express markers of T-cell activation such as programmed death (PD)-1, T-cell immunoglobulin and mucin domain-containing protein (Tim)-3, and lymphocyte activation gene (LAG) 3, which implies continuous stimulation during latency but also functional exhaustion [9,33].

Previously, we demonstrated that the Fas/FasL pathway is involved in HSV-1-induced excessive neuroinflammation within the brain [27]. During primary infection, mice lacking Fas or FasL showed fewer infiltrating inflammatory monocytes and activated microglia in in TGs and brains, while in latency, we observed no differences in the infiltration of cells responsible for neuroinflammation between mice lacking Fas or FasL and wild-type mice. At the same time, a significantly decreased expression of antiviral and inflammatory cytokines and chemokines was detected, implying that despite accumulation, inflammatory monocytes may be less functionally active. On the contrary, when Fas/FasL-deficient mice became infected with HSV-2 via the vaginal route, we observed an opposite clinical situation. Mice lacking Fas or FasL had more severe disease with significantly higher morbidity, mortality, and an overall higher CNS viral load. The immune response to HSV-2 within the spinal cord was impaired, with fewer infiltrating CD4+ T-cells, lower levels of Th1 cytokines and chemokines, and an excessive inflammatory reaction within the spinal cord and genital mucosa [34,35]. We have also demonstrated in vitro that Fas and FasL are required for the induction of leucocyte apoptosis, but also for adequate cytokine and chemokine production by glial cells both during HSV-1 and HSV-2 infection [27,34]. In addition, Morris et al. demonstrated that the presence of FasL on cornea epithelial cells restricts the entry of Fas (+) inflammatory cells and thus reduces the severity of herpetic stromal keratitis (HSK). Mice lacking Fas or FasL showed the most severe clinical HSK [36]. The CNS has been defined as an immune-privileged site protected by the blood–brain barrier (BBB), which is formed by microvascular endothelial cells with tight junctions, pericytes, a basement membrane shared by pericytes and endothelial cells, and astrocytes [37]. Viral encephalitis is accompanied by BBB disruption, enabling the entry of viruses, inflammatory cells, and other immune cells into the brain parenchyma [37]. HSV-1 infection upregulates chemokine receptors, leukocyte adhesion proteins, matrix metalloproteins, and inflammatory markers, affecting the integrity of the BBB [37,38] and leading to further mobilization of peripheral immune cells to the infected sites. Here, we demonstrated that during primary infection, wild-type mice showed an upregulation of claudins and other tight-junction-related proteins (ESAM, JAM-1 and Hcls1) in the infected brains, while a lack of Fas or FasL resulted in much lower upregulation of these genes due to lower neuroinflammation. We have not observed any changes in expression levels of ZO-1 and occludin—proteins participating in tight junctions. Most studies on the neurotropic viruses and their influence upon the expression of tight junction proteins have been performed in vitro using cell lines of endothelial origin, sometimes as 3D models of BBB [39,40]. The studies usually show that viral infection leads to a decrease in the expression of ZO-1, occludin, and claudin proteins, followed by the apoptosis of infected cells, as demonstrated for HSV-1 [39] and Zika virus [40]. However, HSV-1 brain infection precedes BBB disruption (the virus reaches the brain via nerve endings in the brain stem and/or the olfactory bulb) [1,4]. We may presume that disturbances of the BBB are indirectly dependent on the inflammation caused by HSE or directly caused by HSV-1 infection of epithelial cells. The role of claudins, occludin, and ZO-1 in the maintenance of tight junctions during HSV-1 infection is poorly understood. Coronavirus infection of mice with different strains of murine hepatitis virus (MHV) demonstrated that the upregulation of claudin-5 is correlated with BBB leakage and higher pathogenicity of MHV [41]. Zika virus requires the expression of claudin-7 to infect brain endothelial cells [42]. The role of particular proteins present in tight junctions in the maintenance of the BBB during HSV infection may be time- and site-dependent and requires further studies.

The hypothesis that periodic reactivations of HSV-1 after the establishment of latency in the CNS may predispose the brain to AD is now well documented [10,11,12,13,14,15,16,17,18,19]. It is believed that Aβ and tau, together with other proteins involved in neurodegeneration, play a role in the CNS’s innate immune response against different pathogens [10,12]. Previous studies demonstrated signs of neurodegeneration in experimental models of HSV-1 encephalitis [17,18]. Wozniak et al. was the first to demonstrate the presence of Aβ in HSV-1-infected mouse brains at 5 days after intranasal infection [43]. Using a model of a mild and recurrent HSV-1 infection of the CNS, De Chiara et al. documented the accumulation of amyloid-β protein, tau hyperphosphorylation, and neuroinflammation markers (astrogliosis, IL-1β, and IL-6) [17,18]. Here, we observed diffused deposits of Aβ around the HSV-1 infection foci in the primary infection, but later in latency, Aβ was observed as intra- and extracellular aggregates of latently infected neurons. However, the amounts of observed Aβ deposits were less abundant than in the study by De Chiara [17], where several reactivations induced irreversible neurodegeneration. We also found that brain extracts from latently infected mice lacking Fas or FasL contained significantly lower amounts of Aβ and tau hyperphosphorylation compared to wild-type mice. Su et al. reported that AD brains exhibited increased immunoreactivity for Fas and FasL; FasL expression was visibly increased within senile plaques and neurofilament-positive dystrophic neurites and was associated with the active caspase-8 form [25]. Furthermore, Aβ1–42-induced cell death was attenuated in cortical neuron cultures obtained from LPR and GLD mice, suggesting that Fas/FasL death receptor pathways may contribute to Aβ neurotoxicity and AD neurodegeneration [25]. Here, we observed cognitive impairment within the first month of infection in all tested strains, which could result from recent infection, but, eventually, mice lacking Fas or FasL recovered to the cognitive status observed in uninfected, age-matched controls. The fact that a lack of Fas/FasL protected from cognitive deficits is even more important considering the work demonstrating that Fas-deficient (LPR) mice exhibit deficits in learning and memory, measured as the success rates in the T-maze compared to controls. The observed deficits were independent regardless of whether neuronal Fas was mutated in either the immune or nervous system or both, and the authors contributed it to disturbances in neurogenesis in Fas-deficient mice [23]. The involvement of the Fas/FasL pathway in learning and memory may also explain the differences observed for LPR in NOR tests: eventually, the mice recovered from HSV infection, albeit very slowly. Although our study did not include stress, which could lead to HSV reactivation, the wild-type mice showed cognitive impairment after 4 months from infection, correlating with Aβ and tau accumulation. Taken together, our results suggest that a lack of Fas/FasL does not protect from latent infection and virus reactivation, but due to decreased neuroinflammation, it helps to protect from neurodegeneration, although the exact mechanisms remain to be elucidated.

The administration of adjunctive immunomodulatory drugs during the peak of the inflammatory response in HSE patients has been suggested to alleviate possible neurological sequelae and white matter deficits [9]. Glucocorticoids are broad-spectrum anti-inflammatory drugs that bind to the glucocorticoid receptor (GR) and block its interaction with transcription factors such as AP-1 and NF-κB, leading to the suppression of proinflammatory genes. Glucocorticoids can also modulate signal transduction pathways by blocking the interaction of the glucocorticoid receptor with several kinases, such as phosphoinositide 3-kinase (PI3K), mitogen-activated protein kinase (MAPK), and AKT [44]. Several studies evaluated the effect of subcutaneous injections of steroids upon HSV-1 infection [44,45]. The study by Sergerie et al. showed that the delayed administration of glucocorticoids (dexamethasone or corticosterone) initiated at the onset of symptoms (day 3 p.i.) in HSV-1-infected mice decreased the viral titers and cytokine production in the brain, resulting in an increased survival rate [45]. In contrast, early corticosterone treatment started at 0 d p.i. induced an increase in the mortality rate [46]. Ostler et al. demonstrated that GR activation by DEX and stress-induced cellular transcription factors can increase the frequency of reactivation of HSV-1 from latency or viral replication in certain non-neuronal cells (such as epithelial Vero cells) [47]. Dexamethasone was also shown to trigger HSV-1 replication in explants of nasopharyngeal lymphoid tissue (NALT) containing latently infected cells [48]. Therefore, its role in the regulation of neuroinflammation may be complex. Furthermore, there are many reports showing that dexamethasone use in severe COVID cases led to the reactivation of HSV-1 infection [49,50].

In this study, we tested two treatment regimens—during early primary infection and at the peak of the brain infection. As shown previously, early treatment increased mortality and HSV-1 replication in TGs, while later treatment improved clinical outcomes and decreased viral replication in brain parts. An important observation involved TGs, where any kind of treatment increased viral replication, implying that treatment with steroids may worsen the development of peripheral antiviral responses and efficient control of HSV in TGs. Similar effects were maintained throughout the latency, since the virus titers in dexamethasone-treated animals were higher in TGs and lower in latently infected brains. Additionally, dexamethasone treatment facilitated the transcription of the ICP0 protein involved in viral reactivation. DEX-inducible cellular factors in sensory TG neurons were previously identified following the explant of TGs from mice latently infected with HSV-1 [51]. Here, only late treatment with dexamethasone improved antiviral response in brains and increased the infiltration of immune competent cells into the brain. Microglial cells possess GR, and the influence of dexamethasone upon microglia can be dose-dependent; low doses show anti-inflammatory effects, while high doses have been shown to inhibit M2 microglia through the GR/JAK1/STAT3 signaling pathway (pro-inflammatory effect) [52]. Here, we demonstrated that dexamethasone applied during the peak of encephalitis was enough to reduce the excessive infiltration of M1 monocytes and/or to reduce their pro-inflammatory activity.

Unfortunately, dexamethasone treatment during the peak of infection did not protect from the accumulation of Aβ, though it partially protected mice from the accumulation of phosphorylated tau. It should be stressed that the accumulation of Aβ is a long-term process, and apparently closely related with the presence of active HSV replication, leading to constant neuronal loss and eventual cognitive impairment. The presence of HSV-1 has been demonstrated to co-localize with amyloid plaques in AD patients [10,14]. Short-term treatment with dexamethasone was not enough to block HSV–amyloid interaction. On the contrary, the lack of prolonged Fas/FasL-related inflammation and excessive HSV-1 replication provided better protection from the long-term accumulation of beta amyloid than short-term treatment with glucocorticoids. Reducing neuroinflammation during the peak of encephalitis helps to reduce tau levels later in latency by unknown mechanisms.

The Fas receptor can act via non-canonic pathways, triggering signaling pathways such as NF-κB, MAPK, and PI3K/AKT, which, in turn, regulate immune responses and induce the production of inflammatory factors and chemokines [20]. Fas-mediated signaling pathways exhibit considerable crosstalk; thus, the addition of dexamethasone, which may act through similar, non-genomic pathways, seems to add to the Fas-mediated non-apoptotic signaling pathways. Dexamethasone was shown to inhibit the upregulation of Fas and FasL by IFN-γ [53] and we observed here that dexamethasone decreased FasL expression in the cortex and midbrain, therefore helping to balance Fas/FasL-driven inflammatory reactions in HSV-1-infected brain parts. The presence of the Fas/FasL pathway, together with dexamethasone treatment, helped to increase the expression of IFN-γ and -β and IL-6 and to decrease pro-inflammatory IL-1β and TNF-α by HSV-1-infected mixed glial cultures in vitro.

It is generally accepted that treatment with steroids should be initiated as early as possible in CNS infections to avoid neurological sequelae caused by neuroinflammation. However, our study demonstrated that the administration of immunomodulatory agents should be carefully adjusted to the onset of neurological symptoms to ameliorate HSV-1-mediated disturbances of non-apoptotic Fas signaling. Furthermore, it should always be accompanied and preceded by antiviral treatment and a proper selection of dose.

## Data Availability

The original contributions presented in this study are included in the article/Supplementary Materials. Further inquiries can be directed to the corresponding author.

## Supplementary Materials

The following supporting information can be downloaded at https://www.mdpi.com/article/10.3390/cells14241938/s1, Figure S1. Mortality of mice at different time points. Data were presented as mean ± SEM, N = 30. C57BL/6 (WT), B6. MRL-Fas lpr/J (Fas−) and B6Smn.C3-Fasl gld/J (FasL−) mice were infected intranasally with HSV-1, and observed for 4 months. Data analysis was performed by comparing Fas-deficient (lpr) and FasL-deficient (gld) groups with wild-type (C57BL/6) mice. The bars represent means ± SEMs. * *p* ≤ 0.05. Figure S2. Fas/FasL influences infiltration of monocytes during latent HSV-1 infection of TGs. Cell counts for inflammatory monocytes in brains (A), TGs (B) and microglia in brains (C) measured by flow cytometry in mice uninfected and infected at 7 and 120 days p. i. Data were presented as mean ± SEM, N = 7. C57BL/6 (WT), B6. MRL-Fas lpr/J (Fas−) and B6Smn.C3-Fasl gld/J (FasL−) mice were infected intranasally with HSV-1, and at day 7 and 120, brains and TGs were collected for further studies. Data analysis was performed by comparing Fas-deficient (lpr) and FasL-deficient (gld) groups with wild-type (C57BL/6) mice. The bars represent means ± SEMs. **** *p* ≤ 0.001, and * *p* ≤ 0.05. Figure S3. Cytokines and chemokines expressed in brains and TGs of latently infected mice. CXCL9, CXCL10, IFN-γ, TNF-α and IL-1β expression in brain part measured by qPCR. C57BL/6 (WT), B6. MRL-Fas lpr/J (Fas−) and B6Smn.C3-Fasl gld/J (FasL−) mice were infected intranasally with HSV-1 and at day 120, brains and TGs were collected for further studies. Results are expressed as the mean ± SEM for N = 7. ** *p* ≤ 0.01, and * *p* ≤ 0.05 compared to wild-type mice. Table S1. Tight junction-related genes up-regulated or down-regulated in HSV-1 infected brains of C57BL/6 (WT), B6. MRL-Fas lpr/J (Fas−) and B6Smn.C3-Fasl gld/J (FasL−) mice.

## Abbreviations

The following abbreviations are used in this manuscript:

d p.i.	days post infection
DEX	dexamethasone
WT	wild-type C57BL/6
LPR	MRL-Fas lpr/J (Fas−)
GLD	B6Smn.C3-Fasl gld/J (FasL−)
