# Supplementary material for: Lack of Fas/FasL Does Not Protect from Latent Herpes Simplex 1 Infection but Decreases Virus-Induced Neurodegeneration
Source: Cells. 2025 Dec 5;14(24):1938. doi: 10.3390/cells14241938 (PMC12730924; doi:10.3390/cells14241938)
Supplement: Supplementary file 1 [file cells-14-01938-s001.zip › cells-3981488-supplementary.pdf]

Supplementary Materials

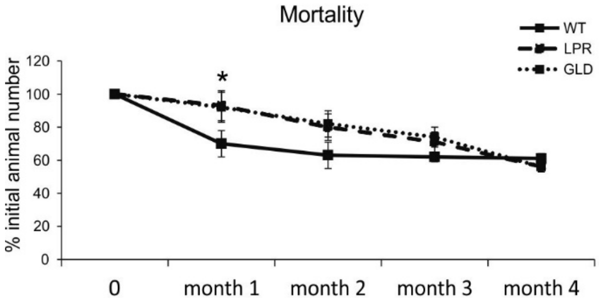

Figure S1. Mortality of mice at different time points. Data were presented as mean ± SEM, N = 30. C57BL/6 (WT), B6. MRL-Fas lpr/J (Fas−) and B6Smn.C3-Fasl gld/J (FasL−) mice were infected intranasally with HSV-1, and observed for 4 months. Data analysis was performed by comparing Fas-deficient (lpr) and FasL-deficient (gld) groups with wild-type (C57BL/6) mice. The bars represent means ± SEMs. *p ≤ 0.05.

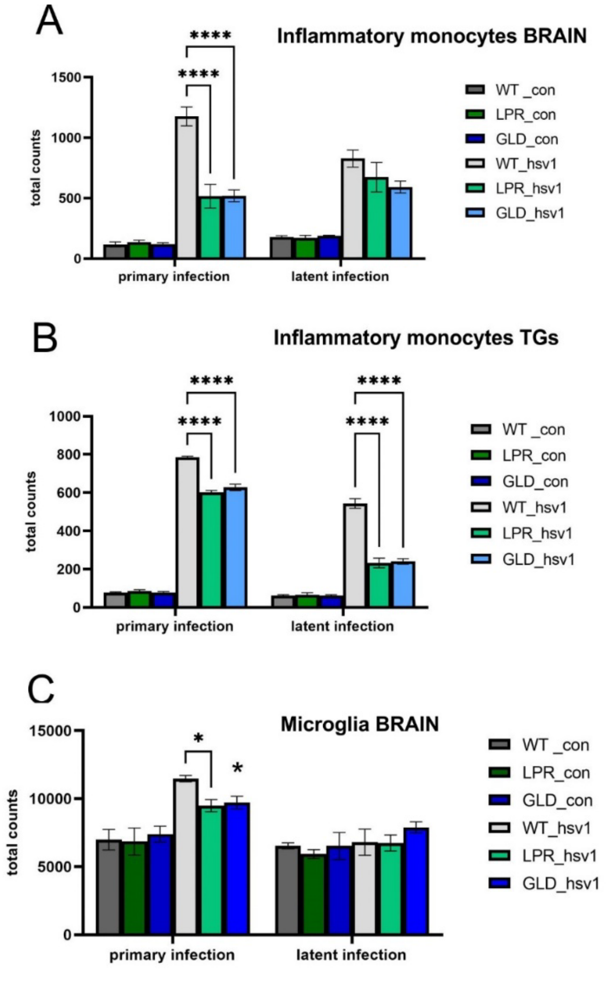

Figure S2. Fas/FasL influences infiltration of monocytes during latent HSV-1 infection of TGs. Cell counts for inflammatory monocytes in brains (A), TGs (B) and microglia in brains (C) measured by flow cytometry in mice uninfected and infected at 7 and 120 days p. i. Data were presented as mean ± SEM, N = 7. C57BL/6 (WT), B6. MRL-Fas lpr/J (Fas−) and B6Smn.C3-Fasl gld/J (FasL−) mice were infected intranasally with HSV-1, and at day 7 and 120, brains and TGs were collected for further studies. Data analysis was performed by comparing Fas-deficient (lpr) and FasL-deficient (gld) groups with wild-type (C57BL/6) mice. The bars represent means ± SEMs. ****p ≤ 0.001, and *p ≤ 0.05.

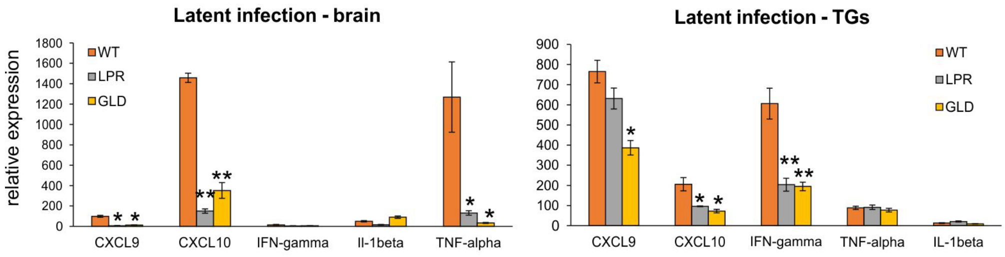

Figure 3S. Cytokines and chemokines expressed in brains and TGs of latently infected mice. CXCL9, CXCL10, IFN-γ, TNF-α and IL-1β expression in brain part measured by qPCR. C57BL/6 (WT), B6. MRL-Fas lpr/J (Fas−) and B6Smn.C3-Fasl gld/J (FasL−) mice were infected intranasally with HSV-1 and at day 120, brains and TGs were collected for further studies. Results are expressed as the mean ± SEM for N = 7. ** $p \leq 0.01$, and *$p \leq 0.05$ compared to wild-type mice.

Table S1. Tight junction-related genes up-regulated or down-regulated in HSV-1 infected brains of C57BL/6 (WT), B6. MRL-Fas lpr/J (Fas−) and B6Smn.C3-Fasl gld/J (FasL−) mice.

Description[1]	Symbol	WT	LPR vs. WT	GLD vs. WT
Claudin 1	Cldn1	*3,5	*-2,76	*-3,08
Claudin 2	Cldn2	2,51	-3,23	-3,38
Claudin 4	Cldn4	*6,1	-*2,63	*-3,49
Claudin 5	Cldn5	**5,48	0	0
Claudin 6	Cldn6	**10,43	*-3,5	-2,2
Claudin 8	Cldn8	2,74	*-3,18	
Claudin 15	Cldn15	**10,03	*-4,3	*-2,88
Claudin 16	Cldn16	**4,88	*-8,86	*-7,36
Claudin 17	Cldn17	2,24	-3,54	-3,02
Claudin 18	Cldn18	2,7	*-23,71	*-14,28
Endothelial cell-specific adhesion molecule	Esam	*3,08	0	0
Hematopoietic cell specific Lyn substrate 1	Hcls1	*3,57	0	0
Intercellular adhesion molecule 1	Icam1	*7,38	0	0

[1] Fold change in tight junction genes expression at 7 day of infection (N=5). Normalization was performed with five housekeeping genes, as described in materials and methods. Expression of gen of interest (GOI) at 7 day of HSV-1 infection was normalized to the expression of the same GOI in the control, uninfected tissue.
